# The locus coeruleus‐dorsal hippocampal CA1 pathway is involved in depression‐induced perioperative neurocognitive disorders in adult mice

**DOI:** 10.1111/cns.14406

**Published:** 2023-08-14

**Authors:** Kai Zhang, Qianqian Chang, Feixiang Li, Yun Li, Ran Ding, Yonghao Yu

**Affiliations:** ^1^ Department of Anesthesiology Tianjin Medical University General Hospital Tianjin China; ^2^ Tianjin Institute of Anesthesiology Tianjin China; ^3^ School of Pharmacy Tianjin Medical University Tianjin China

**Keywords:** chemogenetics, depression, LC‐dCA1 pathway, perioperative neurocognitive disorders, synaptic alterations

## Abstract

**Background:**

Patients undergoing surgical anesthesia increasingly suffer from preoperative depression. Clinical studies have shown that depression is a risk factor for perioperative neurocognitive disorders (PNDs) in elder patients. However, the underlying mechanism, especially at the neural circuit level, remains poorly understood.

**Methods:**

Right carotid artery separation under sevoflurane and chronic social defeat stress (CSDS) in adult mice were used to establish surgical anesthesia and chronic depression models. Cognitive function was assessed by the Y maze and novel object recognition tests. A chemogenetic approach was used to modulate the locus coeruleus‐dorsal hippocampal CA1 (LC‐dCA1) circuit. Hippocampal synaptic alterations were evaluated by Golgi staining and whole‐cell patch clamp recording.

**Results:**

We found that CSDS induced synaptic impairments in dorsal hippocampal CA1 pyramidal neurons and cognitive deficits in adult mice after surgery under sevoflurane. Chemogenetic activation of the LC‐dCA1 pathway significantly alleviated the CSDS‐induced synaptic impairments and cognitive dysfunction. On the contrary, inhibition of this pathway could mimic CSDS‐induced deficits. Furthermore, we showed that dopamine played an important role in CSDS‐induced PNDs in adult mice after surgery/sevoflurane.

**Conclusion:**

Overall, our results have demonstrated a vital role for the LC‐dCA1 pathway in CSDS‐induced PNDs in adult mice undergoing surgery with sevoflurane anesthesia.

## Graphical abstract

Chronic social defeat stress induced synaptic impairments in dorsal hippocampal CA1 pyramidal neurons and cognitive deficits in adult mice after surgery under sevoflurane. Chemogenetic activation of the LC‐CA1 neural pathway ameliorates depression‐induced perioperative neurocognitive disorders through increased release of dopamine.

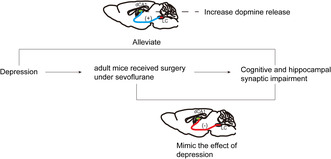

## 1 INTRODUCTION

Perioperative neurocognitive disorders (PNDs), currently including preoperatively diagnosed cognitive decline, postoperative delirium, delayed neurocognitive recovery, and postoperative cognitive impairment, are a common perioperative complication of the central nervous system. It is generally characterized by cognitive impairment, decreased information processing ability and concentration, and accompanied by a series of negative emotions, such as mood and personality changes.^1^ Patients of all ages with surgery and anesthesia may be at risk of PND, with an overall incidence of 2%–3%. However, its incidence can reach 50% in high‐risk patients, such as elderly patients undergoing cardiac and orthopedic surgery.^2^, ^3^, ^4^ PND not only leads to a decrease in patients' quality of life but also significantly correlates with the prognosis of disease exacerbation.^5^ The occurrence of PND may be related to various pathological mechanisms, including impaired synaptic function,^6^ injured blood–brain barrier,^7^ increased neuroinflammation,^8^ mitochondrial dysfunction and enhanced oxidative stress response^9^ and intestinal flora imbalance,^10^ and so on. In addition, PND may also have a common pathogenesis with some neurological and psychiatric diseases. For example, it has been reported that the typical pathological features of Alzheimer's disease (increased Aβ deposition and phosphorylated tau protein) are involved in mediating the occurrence of PND,^11^ and preoperative depression has also been shown to increase the risk of PND.^12^ Therefore, PND is not only a disease state related to the nervous system but also closely related to mental disorders.

Depression is a multifactorial disorder characterized by depressed mood, anhedonia, difficulty thinking and making decisions, appetite, sleep, psychomotor disturbances, and even suicidal ideation.^13^ The World Health Organization has listed major depressive disorder as the fourth leading cause of disability worldwide and predicted it would become the second leading cause of disability by 2030.^14^

Especially after the outbreak of the COVID‐19 epidemic, the number of patients with depression has soared. During this period, the number of newly emerging depressed patients worldwide has exceeded 70 million according to the report of the World Health Organization in 2021.^15^ In addition, a questionnaire survey in the United States (Mental Health America Screening) showed that the incidence of depression was 8.5% before the epidemic, but the incidence of depression in 2020 and 2021 climbs to 27.8% and 32.8%, respectively.^16^

With the significant improvement in the medical care, the life expectancy of human beings continues to increase, and there is the annual increase in the cases of patients with depression receiving surgery and anesthesia. Previous clinical studies have shown that preoperative anxiety and depression are the independent risk factors for postoperative delirium, and the degree of depression significantly correlates with the severity and duration of delirium.^17^, ^18^ However, the mechanisms by which depression induces postoperative cognitive impairment are poorly understood. We aim to elucidate the mechanisms underlying depression conferring the susceptibility of cognitive impairment induced by surgery/anesthesia.

The locus coeruleus (LC) is the primary nucleus of norepinephrine production in the brain, which has many neural circuit connections with multiple regions in the brain and spinal cord. It regulates cognition, wakefulness, pain and stress, and other physiological functions.^19^ Notably, it has been reported to be linked to the pathogenesis of stress‐related disorders, including depression.^20^, ^21^ For example, chronic stress exposure typically leads to decreased activity of the LC neurons and subsequent reduction in norepinephrine release.^22^ Moreover, it is well established that LC activity is also involved in cognitive behaviors due to its abundant projections to the dorsal hippocampal CA1 (dCA1) region. However, this effect is mediated by dopamine rather than norepinephrine.^23^ Therefore, since individuals with surgery/anesthesia are more vulnerable to cognitive impairments and dCA1 synaptic dysfunction,^24^ we hypothesized that the LC‐dCA1 pathway might mediate the connections between surgery/anesthesia‐induced PND and depression. In addition, we would like to further determine whether dopamine or norepinephrine mediates these effects.

In this study, adult mice were subjected to chronic social defeat stress (CSDS) prior to surgery/sevoflurane. We found that cognitive function was impaired in mice susceptible to CSDS, as reflected by poor behavioral performance and the impaired synaptic alterations of dCA1 pyramidal neurons. Then, we demonstrated that the bidirectionally chemogenetic regulation of the LC‐dCA1 pathway resulted in opposite responses to surgery/anesthesia. Finally, we further discovered that CSDS‐induced PND in adult mice with surgery/sevoflurane might be mainly due to a decrease in dopamine release. Overall, our results elucidate the role of the LC‐dCA1 pathway in depression‐associated cognitive dysfunction after surgery/sevoflurane in adult mice.

## 2 MATERIALS AND METHODS

### 2.1 Animals

Male C57BL/6J mice (weighing 20–25 g) around 10 weeks old were used for this experiment and randomly grouped. Three or four mice were raised in each cage except for those mice implanted with optical fibers caged alone. The animals were freely given access to water and food. They were kept under the condition of a half‐day light–dark cycle (lights on at 7:10 a.m., standard Beijing time) at basically unchanged temperature (23 ± 2°C) and humidity (50% ± 10%). All behavioral tasks were conducted during the light period. All the experimental procedures were conducted according to the “Guide for the Care and Use of Laboratory Animals” published by the National Institutes of Health (NIH) and approved by the Animal Care and Use Committee of Tianjin Medical University of Traditional Chinese Medicine.

### 2.2 Chronic social defeat stress

CSDS protocol was conducted according to the previously described model.^25^ Before the experiment, CD1 mice needed to be screened on three successive days and selected based on the following criteria: (1) latency of attack less than 60 s and (2) attacking for two consecutive days. For 10 days, experimental C57BL/6J mice were forced to interact physically and defeated by the aggressive CD1 mice in the resident home cage for 5–10 min per day at 3:00–6:00 p.m. (Beijing Standard Time). After the physical defeat, an iron fence divider was used to divide the C57BL/6J mice and CD1 mice in a home cage to allow visual, auditory, and olfactory interaction between them for 24 h until the physical defeat in next day. The C57BL/6J mice faced novel CD1 mice every day. The control mice were housed together, separated by the iron fence divider barrier, and switched each day, and the CD1 mice never defeated them. After 10 days of CSDS, the experimental mice were housed alone and tested 24 h later for depressive‐like behaviors.

### 2.3 Surgery and anesthesia model

**FIGURE 1:**
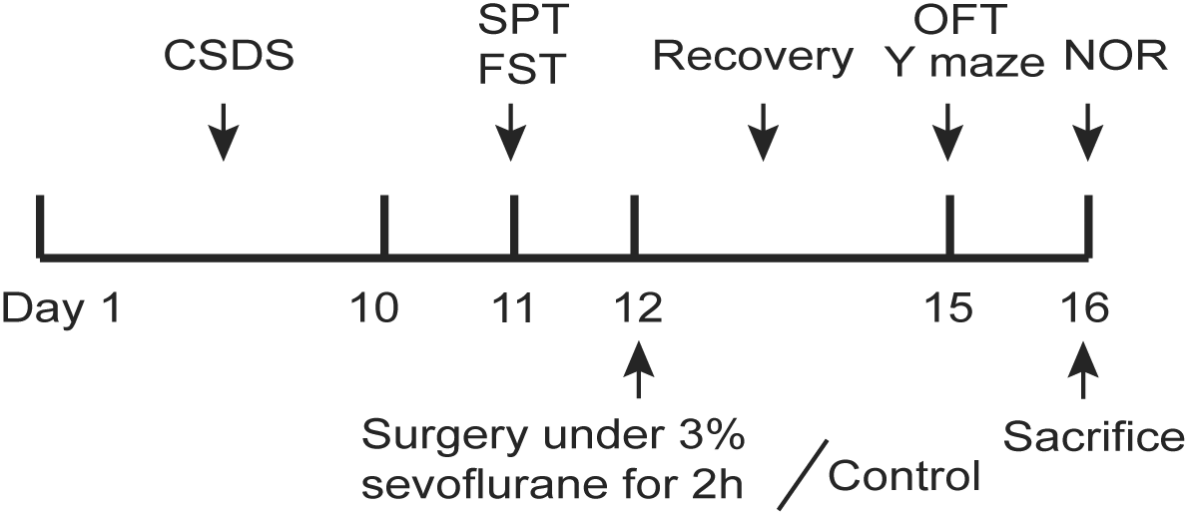
Schematic diagram of experimental procedures.

We performed right carotid artery separation and exposure under sevoflurane inhalation to establish a surgical anesthesia model. The surgery was performed 2 days after the end of CSDS (Figure 1). The day before surgery, mice were housed in the operating room to adapt to the environment. Mice were anesthetized with a mixed gas (3% sevoflurane and 2 L/min pure O_2_). The body temperature was kept at 37 ± 0.5°C using a heating pad during the procedure. After confirming that the mouse did not respond to the pain stimulus, a 2.5 cm long incision was made longitudinally on one side of the mouse's neck after being disinfected with iodine. The soft tissue was then gently dissected with forceps under a microscope until the common carotid artery was exposed. After gently separating the common carotid artery and surrounding tissue, the skin was sutured and disinfected again. Then, the mice continued to be maintained anesthesia inhalation under 3% sevoflurane, and the total time of surgery plus subsequent anesthesia inhalation was 2 h. Finally, the mice were transferred to a warm environment for recovery for approximately 2 h before being back to their cages.

### 2.4 Viral vectors

AAV2/9 viruses encoding Ef1a‐DIO‐hM3Dq‐mCherry‐WPREs (2 × 10^12^ vg/mL), Ef1a‐DIO‐hM4Di‐mCherry‐WPREs (2 × 10^12^ vg/mL, 150 nL/injection site) and Ef1a‐DIO‐mCherry‐WPREs‐hGH ploA (2 × 10^12^ vg/mL, 150 nL/injection site), retrograde virus AAV/Retro‐hSyn‐cre‐GFP‐WPREs (5 × 10^12^ vg/mL, 150 nL/injection site) were used for chemogenetic modulation of the LC‐dCA1 pathway. In addition, two kinds of neurotransmitter probes viruses were used: pAAV‐hSyn‐DA3m‐WPREs (2 × 10^12^ vg/mL, 200 nL/injection site) and pAAV‐hSyn‐GRAB‐NE(2 m)‐WPREs‐pA (2 × 10^12^ vg/mL, 200 nL/injection site) for monitoring changes in the concentration of neurotransmitters. All the viruses above were purchased from BrainVTA (BrainVTA Co., Ltd.). Viral vectors were equally subdivided into 2 μL stored at −80°C until use.

### 2.5 Stereotaxic surgery

Mice were anesthetized with a mixed gas (1.2% isoflurane and 0.5 L/min air) and placed in a stereotaxic apparatus (68030, RWD, China). Body temperature remained at 37 ± 0.5°C using a heating pad. About 20 μm in tip diameter of a glass micropipette was used to inject the virus via a small skull opening (<0.5 mm^2^). After each injection, the syringe was left for 10 min before being withdrawn to ensure the virus fully diffused. Mice recovered on a heating pad until normal behavior resumed. All experiments involving viral constructs were conducted for at least after 3 weeks to make sure sufficient expression of viruses and recovery. Viral infusion coordinates were dCA1 (from bregma: anterior–posterior (AP), −1.8 mm, mediolateral (ML), ±1.5 mm, and dorsal–ventral (DV) from the dura, −1.25 mm) and the LC (from bregma: AP, −5.3 mm, ML, ±0.9 mm, and DV from the dura, −4.0 mm). Both the LC and dCA1 received bilateral injections.

### 2.6 In vivo optical fiber‐based neurotransmitter probe signaling recordings

For probe signal recording, the dCA1 received a unilateral injection of 200 nL pAAV‐hSyn‐DA3m‐WPREs or pAAV‐hSyn‐GRAB‐NE(2 m)‐WPREs‐pA at a rate of 50 nL/min. A custom‐built device was applied for neurotransmitter probe signal measurements (Thinker Tech Nanjing Bioscience Inc). A fiber photometry system, including one light‐emitting diode at 488 nm, was used as an excitation laser. To minimize the bleaching effect, the fiber tip's light intensity was adjusted to ∼0.22 μW/mm^2^. We used a black fiber to guide the light between the fiber photometry system and the implanted optical fiber on the mice's skulls. Probe signals were digitized at 2000 Hz with written data acquisition software based on LabVIEW (National Instruments; Suzhou Institute of Biomedical Engineering and Technology). All probe signals and behavioral videos were synchronized offline with event marks.

### 2.7 Golgi staining and sholl analysis and spines counting

Mice brains (three mice per group) were used for Golgi impregnation using the FD Rapid Golgi Stain Kit (FD NeuroTechnologies). The tissues were coronally cut into a thickness of 100 μm with a vibratome. After overnight, all slices were sequentially stained according to the manufacturer's procedure and put in darkness. A single person examined pyramidal cells from dCA1 neurons and subsequent counts. We used a 100× oil objective on an upright light microscope to observe dCA1 neurons, and six cells were randomly chosen from an individual animal for further analysis. Sholl analysis was performed as described previously.^26^ In brief, dendritic trees were reconstructed with the tracing tool, and their complexity was analyzed by 8‐bit images using ImageJ (Open Source from NIH, Bethesda, MD). We quantified the number of intersections of branches with the concentric circles of increasing radius plotted around the center of pyramidal soma. The starting radius, ending radius, and the interval between consecutive radii were 10 μm (from the center), 160 μm, and 10 μm, respectively. In addition, spines on apical secondary dendrites were counted. Quantitative analysis was performed using the ImageJ software.

### 2.8 Patch‐clamp electrophysiological recording of brain slices

As described previously,^27^ mice were decapitated quickly under deep anesthesia and the brains were immediately transferred into ice‐cold oxygenated (95%O_2_ + 5%CO_2_ mixture) artificial cerebrospinal fluid (ACSF) for 1 min. The ACSF (in mM) includes 120 NaCl, 2.5 KCl, 2 CaCl_2_·2H_2_O, 1.25 KH_2_PO_4_, 2 MgSO_4_·7H_2_O, 26 NaHCO_3_, and 10 glucose. Then, the brain tissue containing dCA1 was sectioned coronally at a 300 μm thickness in the same buffer using a vibratome (LEICA, CM1950). Next, they were incubated in oxygenated ACSF at 33°C for 30 min followed by oxygenated ACSF at room temperature for at least 1 h before recording. To record spontaneous excitatory postsynaptic current (sEPSC) of dCA1 pyramidal neurons, the whole cell patch clamp was recorded under voltage clamp at −60 mV. Picrotoxin (100 μM) was applied to block γ‐aminobutyric acid (GABA‐A) receptors throughout the recording. Pyramidal cells were identified by their pyramidal shape, immense soma, and apical dendrites. The brain section was continuously perfused with the oxygenated ACSF. Electrodes with a resistance of 3–5 MΩ were pulled with a Sutter micropipette puller (P‐97, Novato). Their tips were filled with a solution (in mM, pH 7.25) containing 140 K‐gluconate, 0.1 CaCl_2_, 2 MgCl_2_, 1 ethylene glycol tetraacetic acid (EGTA), 2 ATP, 0.1 GTP·Na3, and 10 N‐(2‐ hydroxyethyl) piperazine‐N′‐(2‐ethane sulfonic acid) (HEPES). Current signals were recorded by an Axon patch 700A amplifier connected to a digital 1322A interface. Each cell was allowed to record no more than two sessions with each 5 min duration. The data were digitized and stored on disks using Clampfit (version 8.2.0.228; Axon).

The spontaneous action potentials of LC neurons induced by Clozapine‐N‐oxide (CNO, 10 μM) was recorded using a current clamp under the cell‐attached mode. CNO was added into ACSF after 5 min of baseline recording and washed after another 5 min. The internal solution of electrodes contained (in mM) 140 KCl, 0.5 EGTA, 5 mgATP, and 10 HEPES, pH = 7.3, 295–300 mmol. The data were also analyzed by Clampfit software.

### 2.9 Behavioral tests

All the mice were subjected to the following behavioral tests, the test procedures are shown in Figure 1. The sucrose preference test (SPT) was from 8:00 a.m. to 8:00 p.m. on the eleventh day, and the FST was performed 1 h later. In addition, the time interval between open field test (OFT) and Y maze tests was also an hour.

#### 2.9.1 Mice weight measure

The body weight of mice was recorded across the whole 10 days of CSDS.

#### 2.9.2 Sucrose preference test

In order to make mice adapt, two bottles of 1% sucrose solution were given to the taste of sucrose 2 days prior to testing. Twenty‐four hours later, one bottle was replaced with tap water for another 24 h. Then after the mice were deprived of a water supply for 12 h, each mouse was given a bottle of 1% sucrose solution and a bottle of tap water with the same volume for 12 h. At the sixth hour, the positions of the two bottles are changed once to avoid location preference. The weight of each bottle was recorded after 12 h, percentage of sucrose preference (%) = (sucrose solution consumption)/(sucrose solution consumption + water consumption) ×100%.

#### 2.9.3 Forced swimming test (FST)

Mice were placed singly into a glass cylinder (diameter 15 cm, height 30 cm) filled with 15 cm depth of water (23 ± 1°C). They were placed in the cylinder for 6 min, and the immobility time was analyzed during the last 4 min.

#### 2.9.4 Y maze test

The Y maze (ZS Dichuang, China) test was applied to assess working memory in mice. The three arms of the device extend out at the same angle from the center. Each arm's length, width, and height are 30, 6, and 20 cm. The three arms were randomly divided into the start arm, the other arm, and the novel arm. The Y‐maze experiment is performed in two steps: training and test phases. Mice were habituated in the experimental room for 1 day before the training phase. Then, they were allowed to explore the Y maze with one arm closed (novel arm) during the 10‐min training phase. After that, mice were removed from their cage and replaced in the Y maze 1 h later (with all arms open) for the 5‐min test phase. After each training or test, the maze was wiped with 75% ethanol to eliminate the smell of the last mouse. Because rodents prefer to explore novel environments, the greater time spent in the novel arm represents an estimate of the reference spatial memory of mice.

#### 2.9.5 Novel objective recognition (NOR) test

A NOR test was used to assess rodents’ spatial learning and memory. Briefly, mice were acclimated to the testing room and empty box (50 cm × 50 cm × 50 cm) for 10 min 1 day before the experiment. The following day, they were permitted to explore two identical objects for 10 min in the box. After 2 h, one of the objects was replaced by a novel object with different colors and shapes. Mice were permitted to explore the two different objects (familiar object and novel object) for 5 min. After every test, the chamber and objects were thoroughly cleaned with 75% ethanol. The novel object preference ratio was analyzed as the time mice spent exploring the novel object divided by the time they spent exploring both objects.

#### 2.9.6 Open field test

A square open field area (50 cm × 50 cm × 50 cm) was used to assess spontaneous activity. The bottom of the box is evenly divided into 16 grids, the central four grids are the central area, and the remaining 12 are the surrounding area. After adapting to the testing room for 1 day, mice were tested for 5 min in the box. The total distance and time spent in the center area were automatically calculated.

#### 2.9.7 Behavioral pharmacology

For microinfusions, we bilaterally injected 0.5 μL β‐adrenoceptor antagonist propranolol (295.80 g/mol, Sigma‐Aldrich) or dopamine D1/D5 receptor antagonist SCH23390 hydrochloride (324.24 g/mol; Tocris) into dCA1 region at the rate of 0.2 μL/min. The concentrations of propranolol and SCH23390 are 21.1 mM (6.25 μg/μL) and 3.1 mM (1 μg/μL), respectively.

### 2.10 Histology and immunofluorescence staining

After completion of experiments, viruses’ expression and fiber position was confirmed by histology. Animals were deeply anesthetized and transcardially perfused with 0.9% saline, followed by 4% paraformaldehyde in 0.1 M PBS. Next, the brains were removed and fixed in 4% paraformaldehyde for at least 24 h at 4°C. Tissue coronal sections (30 μm) of the brain containing the hippocampal dCA1 and LC regions were cut and prepared via a cryostat microtome (LEICA, CM1950) for histology and immunofluorescence staining. Images were captured through confocal microscopy Zeiss 800. For dopamine, D1/D5 receptor staining, the sections containing dCA1 were blocked in PBS solution (pH 7.4, including 5.0% normal goat serum with 0.3% TritonTM X‐100) for 1 h at room temperature. After the goat serum was wiped, the sections (10 μm) were incubated with a primary rabbit anti‐dopamine D1 (1:50, EPR24102‐105, Abcam) overnight at 4°C, followed by a three times 10 min wash with PBST (PBS with 0.1% Triton X‐100, vol/vol). Then, the slices were incubated with goat anti‐rabbit secondary antibody (Alexa 488, 1:500 dilution) at room temperature for 1 h in the dark. Next, after washing twice with PBST for 5 min, slices were incubated in DAPI (Sigma‐Aldrich, 1:1000) for 5 min. DAPI was washed off three times. At last, the sections were cover‐slipped with a fluorescent mounting medium and stored at 4°C before analysis. Images were captured through a fluorescence microscopy Olympus BX51.

### 2.11 Statistical analysis

We analyzed fiber data using a procedure similar to the previous work.^28^ The changes of relative fluorescence DF/F0 = (f–fbaseline)/fbaseline were represented as probe signals, where the fbaseline was the basal level of fluorescence calculated during the whole recording period. A probe signal was accepted when its amplitude was three times the standard deviation of the control time. Data analysis was conducted using Prism 9 (GraphPad Software Inc.). All data were subject to tests for normality using the Shapiro–Wilk test and the Kolmogorov–Smirnov test. Data that did not exhibit a normal distribution were analyzed via the non‐parametric equivalent. Data expression and analysis methods were indicated in each figure legend.

## 3 RESULTS

### 3.1 CSDS‐induced PND and synaptic impairments in adult mice

**FIGURE 2:**
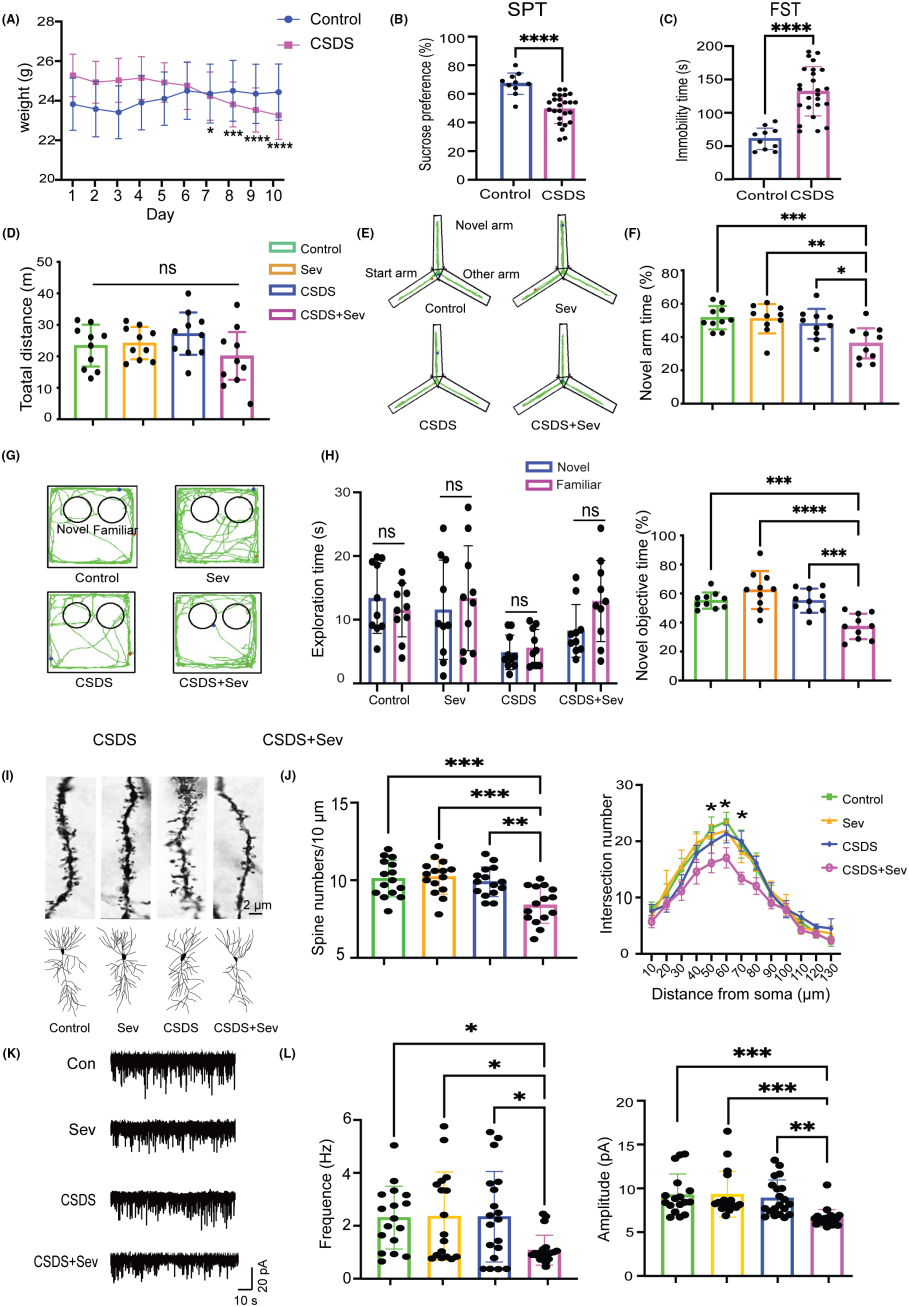
CSDS induced PND and hippocampal synaptic alterations impairment in adult mice after surgery/sevoflurane. (A) The weight of Control (*F*(9) = 1.643, *p* = 0.061) and CSDS (*F*(9) = 17.6, *p* < 0.0001) mice across 10 days (RM one‐way ANOVA, *n* = 10/group). (B) Sucrose preference in SPT test (*p* < 0.0001, unpaired Student's *t*‐test, *n* = 10/control, *n* = 25/CSDS). (C) Immobility time in FST test (*p* < 0.0001, unpaired Student's *t*‐test, *n* = 10/control, *n* = 25/CSDS). (D) Total distance in the OFT test (*F*(3)=2.362, *p* = 0.0849, one‐way ANOVA test, *n* = 10/group). (E) Representative mice track in Y maze test. (F) Percentage of novel arm exploration time in Y maze test (*F*(3) = 8.344, *p* = 0.0002, one‐way ANOVA with Tukey's post hoc test, *n* = 10/group). (G) Representative mice track in NOR test. (H) Exploration time of the two objects in the training phase (left, unpaired Student's *t*‐test and Mann–Whitney U test) and percentage of novel object exploration time in the test phase (right *F*(3) = 13.09, *p* < 0.0001, one‐way ANOVA with Tukey's post hoc test, *n* = 10/group) of the NOR test. (I) Example of spines (top) and dendrites (bottom) of four groups. (J) Statistics of spines numbers (left, *F*(3) = 8.693, *p* < 0.0001) and dendrites (right, *F*(3) = 3.919, *p* = 0.0357) of four groups (one‐way ANOVA with Tukey's post hoc test, *n* = 15 neurons from 3 mice/group). (K) Example of sEPSC for each group. (L) Statistics of frequency (left, *F*(3) = 4.416, *p* = 0.0067) and amplitude (right, *F*(3) = 8.018, *p* = 0.0001) of sEPSC in four groups (one‐way ANOVA with Tukey's post hoc test, *n* = 16 neurons from four mice/group). *p < 0.05, **p < 0.01, ***p < 0.001, ****p < 0.0001. Data are presented as mean ± SD.

To investigate whether repeated stress increased the risk of PND in adult mice with surgery/sevoflurane, mice were subjected to ten consecutive days of CSDS treatment, and then the right carotid artery separation and exposure under sevoflurane inhalation model (Sev) that explicitly affects cognitive function in juvenile^29^ and aged mice.^30^ The weight of control mice remained stable over 10 days, while the weight of CSDS mice steadily decreased (Figure 2A). Moreover, CSDS mice exhibited markedly decreased sucrose preference in SPT (Figure 2B) and increased immobility in FST (Figure 2C) compared to control mice. Both CSDS and Sev did not alter the mice motor locomotion as reflected by the total distance in the OFT test (Figure 2D). However, compared to the other three groups, mice in the CSDS+Sev group displayed lower exploration time both in the novel arm in the Y maze test (Figure 2E,G) and novel object in the NOR test (Figure 2F,H). These results demonstrated that surgery/sevoflurane in adult mice subjected to CSDS led to aggravated vulnerability to learning and memory deficits.

Then, we investigated whether Sev mice subjected to CSDS treatment exhibited an increased risk of hippocampal synaptic impairments, closely related to cognitive performance. As indicated in cognitive behavioral performance, only mice in the CSDS+Sev group showed apparent decreased spine density and complexity in hippocampal dCA1 pyramidal neurons (Figure 2I,J). Furthermore, significantly reduced sEPSC frequency and amplitude were detected in dCA1 neurons in the CSDS+Sev group (Figure 2K,L). Taken together, these data indicated that depression might serve as a potential risk factor in CSDS‐induced PND after surgery/sevoflurane in adult mice.

### 3.2 Activation of the LC‐dCA1 projection rescued CSDS‐induced PND in adult mice

**FIGURE 3:**
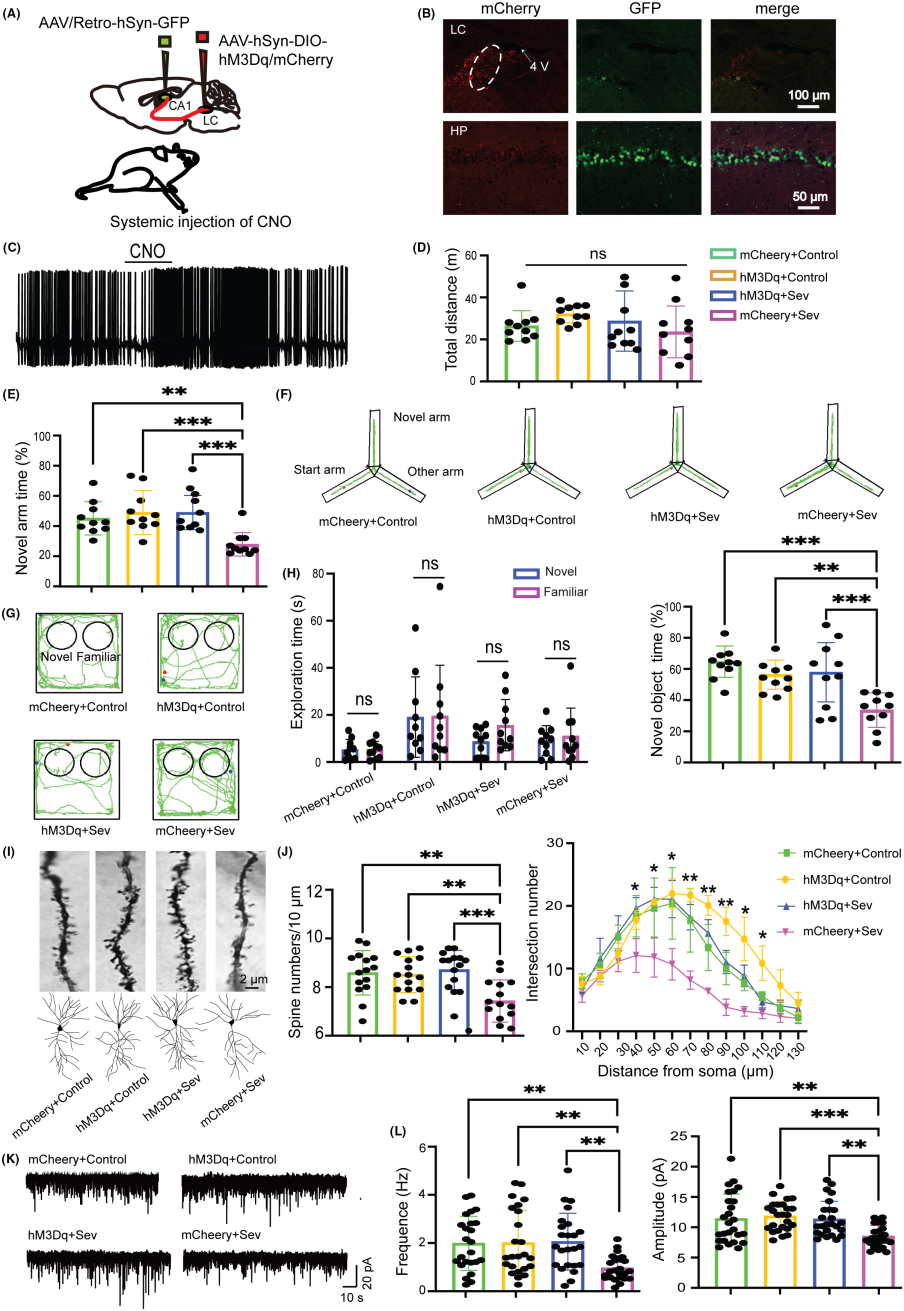
Activation of the LC‐dCA1 pathway improved cognitive function and hippocampal synaptic alterations. (A) Diagram of viruses injection sites. (B) Representative images of viruses expression in the LC (top) and hippocampus (bottom). (C) Example of action potentials of the LC neurons before and after CNO administration. (D) Total distance in the OFT test (*F*(3) = 1.987, *p* = 0.1332). (E) Percentage of novel arm exploration time in Y maze test (*F*(3) = 8.522, *p* = 0.0002). (F) Representative mice track in Y maze test. (G) Representative mice track in NOR test. (H) Exploration time of the two objects in the training phase (left, unpaired Student's *t*‐test and Mann–Whitney U test) and percentage of novel object exploration time in the test phase (right, *F*(3) = 8.711, *p* < 0.001) of the NOR test. (I) Example of spines (top) and dendrites (bottom) of four groups. (J) Statistics of spines numbers (left, *F*(3) = 7.599, *p* < 0.001) and dendrites (right, *F*(3)=25.488, *p* < 0.001) of four groups. (K) Example of sEPSC for each group. (L) Statistics of frequency (left, *F*(3) = 6.171, *p* < 0.001) and amplitude (right, *F*(3) = 6.791, *p* < 0.001) of sEPSC in four groups. One‐way ANOVA with Tukey's post hoc test, *p < 0.05, **p < 0.01, ***p < 0.001, *n* = 10/group. Data are presented as mean ± SD.

Since the LC is involved in both stress and cognitive function, we wondered whether enhancement of the LC‐dCA1 pathway could rescue the adverse effects caused by CSDS on cognitive and hippocampal neuronal synaptic alterations. Thus, we bilaterally injected AAV/Retro‐hSyn‐cre‐GFP‐WPREs and AAV‐Ef1a‐DIO‐hM3Dq‐mCherry or control virus AAV‐Ef1a‐DIO‐mCherry into dCA1 and LC, respectively (Figure 3A). Mice were subjected to CSDS 20 days after viruses injection. And then the animals received 2 mg/kg CNO (1 mg/mL, i.p.) 1 h prior to Sev to guarantee the enhancement of the LC‐dCA1 projections specifically. The viruses were accurately expressed in both the LC and dCA1 regions (Figure 3B), and CNO (10 μM) administration significantly increased the action potentials of the LC neurons in hM3Dq mice (Figure 3C). The four groups, mCherry+Control, mCherry+Sev, hM3Dq + Control, and hM3Dq + Sev were subjected to cognitive behavioral tests and dCA1 neuronal synaptic alterations assessment.

There was no significant difference among the four groups in total distance traveled in the OFT test (Figure 3D). The hM3Dq + Sev group explored more time in the novel arm (Figure 3E,F) and novel object (Figure 3G,H) compared with mCherry+Sev mice in the Y maze and NOR test respectively, while showing no difference compared with mCherry+Control mice. Consistent with the improved spatial learning and memory in hM3Dq + Sev mice, Golgi staining results showed that spine numbers and complexity of dCA1 pyramidal neurons were prominently increased in hM3Dq + Sev mice compared with mCherry+Sev mice (Figure 3I,J). Moreover, the sEPSC frequency and amplitude of dCA1 neurons in hM3Dq + Sev mice were also higher than that in mCherry+Sev mice (Figure 3K,L).

### 3.3 Inhibition of LC‐dCA1 partially mimicked the effects of CSDS‐induced PND

**FIGURE 4:**
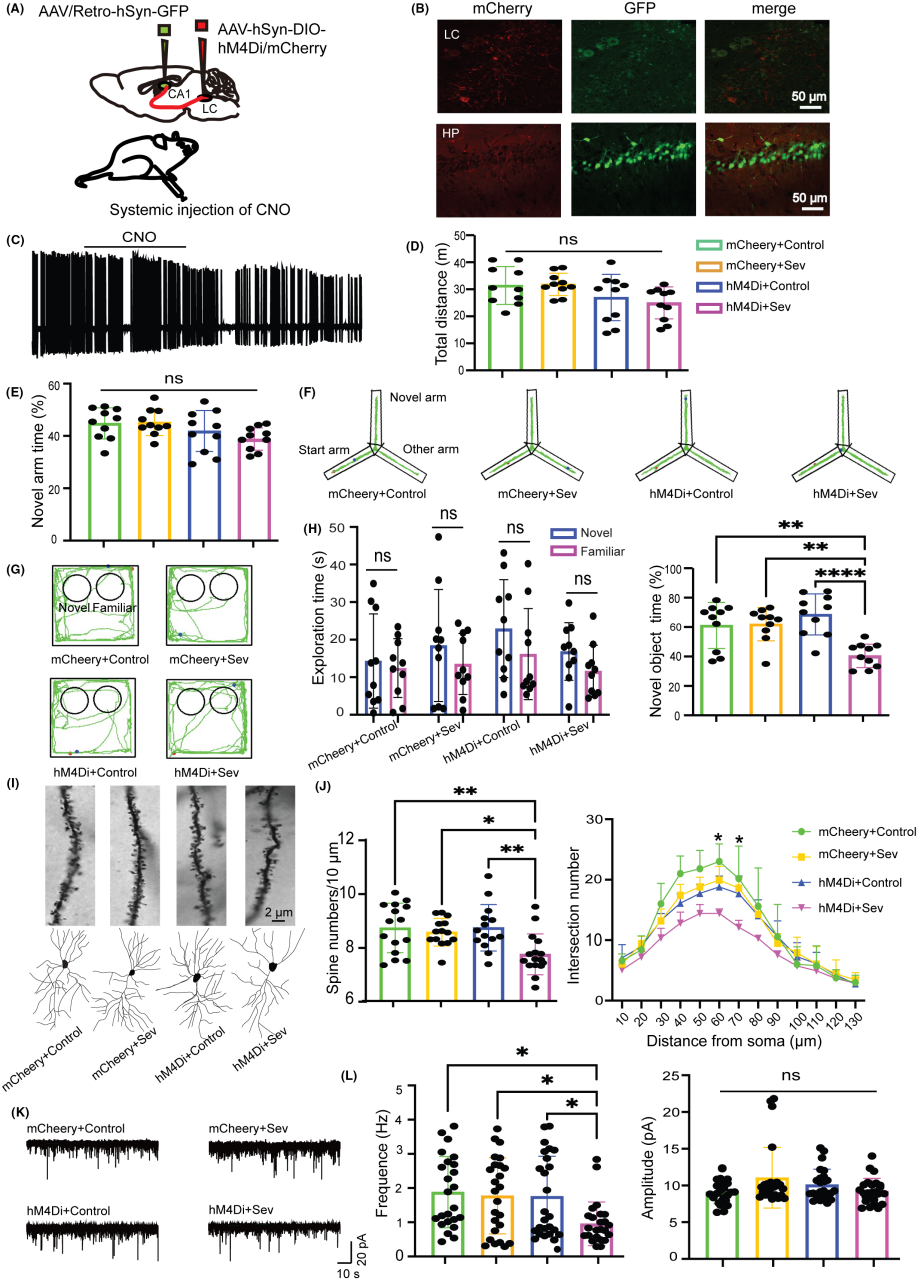
Inhibition of the LC‐dCA1 pathway induced impairments of cognitive and hippocampal synaptic alterations. (A) Diagram of viruses injection sites. (B) Representative images of viruses expression in the LC (top) and hippocampus (bottom). (C) Example of action potentials of the LC neurons before and after CNO administration. (D) Total distance in the OFT test (*F*(3) = 2.702, *p* = 0.06). (E) Percentage of novel arm exploration time in Y maze test (*F*(3) = 2.677, *p* = 0.0607). (F) Representative mice track in Y maze test. (G) Representative mice track in NOR test. (H) Exploration time of the two objects in the training phase (left, unpaired Student's *t*‐test and Mann–Whitney U test) and percentage of novel object exploration time in the test phase (right, *F*(3) = 10.21, *p* < 0.0001) of the NOR test. (I) Example of spines (top) and dendrites (bottom) of four groups. (J) Statistics of spines numbers (left, *F*(3) = 5.644, *p* = 0.0019) and dendrites (right, *F*(3) = 9.628, *p* = 0.0082) of four groups. (K) Example of sEPSC for each group. (L) Statistics of frequency (left, *F*(3) = 4.411, *p* = 0.006) and amplitude (right, *F*(3) = 1.580, *p* = 0.1995) of sEPSC in four groups. One‐way ANOVA with Tukey's post hoc test, *p < 0.05, **p < 0.01, ****p < 0.0001, *n* = 10/group. Data are presented as mean ± SD.

To further validate the LC‐dCA1 projections were required in CSDS‐induced hippocampal vulnerability and PND after surgery/sevoflurane, we used the inhibitory DREADDs to specifically silence the LC‐dCA1 pathway by bilaterally injecting the AAV/Retro‐hSyn‐cre‐GFP‐WPREs and AAV‐Ef1a‐DIO‐hM4Di‐mCherry or AAV‐Ef1a‐DIO‐mCherry into dCA1 and LC, respectively (Figure 4A). Four weeks after the viruses injection, mice were subjected to Sev with 2 mg/kg CNO (1 mg/mL, i.p) injection 1 h before Sev. The viruses were accurately expressed in both the LC and dCA1 regions (Figure 4B), and CNO (10 μM) administration significantly decreased the action potentials of hM4Di expressed in the LC neurons (Figure 4C). Administration of inhibitory DREADDs did not affect the locomotion of mice as indicated in the total distance traveled in the OFT test (Figure 4D).

In the subsequent cognitive behavioral tests, our results demonstrated that hM4Di + Sev mice exhibited less exploration time to novel objects in the NOR test (Figure 4G,H) but not significant difference in exploring novel arm in the Y maze test compared with mCherry+Sev mice (Figure 4E,F). However, both spine numbers and complexity of dCA1 pyramidal neurons dramatically decreased in hM4Di + Sev mice compared to mCherry+Sev mice (Figure 4I,J). Moreover, compared to the mCherry+Sev group, the sEPSC frequency but not amplitude in dCA1 neurons declined in hM4Di + Sev mice (Figure 4K,L). These results indicated that specific inhibition of the LC‐dCA1 circuit could mimic the effects of CSDS‐induced PND after surgery/sevoflurane.

### 3.4 Chemogenetic activation of the LC‐dCA1 pathway rescued CSDS‐induced PND via the Dopamine D1/D5 Receptor

**FIGURE 5:**
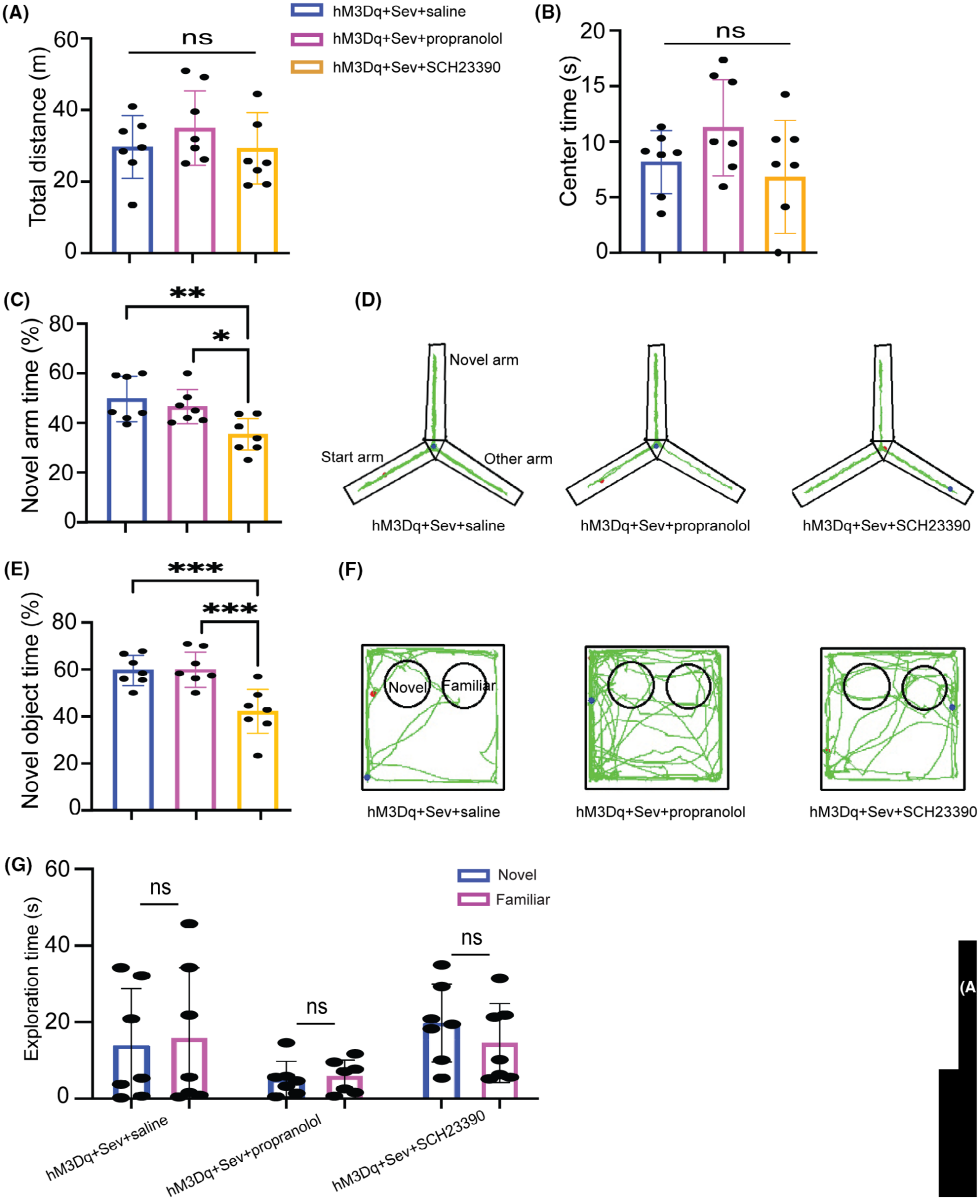
Dopamine served as an important role in the beneficial effect of activating the LC‐dCA1 pathway. (A) Total distance in the OFT test (*F*(2) = 0.8185, *p* = 0.4554). (B) Time spent in the center area in the OFT test (*F*(2) = 2.294, *p* = 0.1268). (C) Percentage of novel arm exploration time in Y maze test (*F*(2) = 8.267, *p* = 0.0024). (D) Representative mice track in Y maze test. (E) Percentage of novel object exploration time in the test phase of the NOR test (*F*(2) = 13.69, *p* < 0.001). (F) Representative mice track in NOR test. (G) Exploration time of the two objects in the training phase of the NOR test (unpaired Student's *t*‐test). One‐way ANOVA with Tukey's post hoc test, *p < 0.05, **p < 0.01, ***p < 0.01, *n* = 7/group. Data are presented as mean ± SD.

LC neurons projecting to the dorsal hippocampus release both norepinephrine and abundant dopamine. We next explored which neurotransmitter mediates the cognitive effects of the LC‐dCA1 pathway in CSDS‐induced PND in adult mice with surgery/sevoflurane. To address this question, after AAV‐hM3Dq expressed sufficiently in the dCA1, we bilaterally infused one of three compounds into the dCA1 region immediately prior to the Sev administration: (1) SCH23390, a dopamine D1/D5 receptor antagonist; (2) propranolol, a beta‐adrenergic receptor antagonist; or (3) saline. As indicated in the total distance traveled (Figure 5A) and time spent in the center area (Figure 5B) in the OFT, there were no significant differences in locomotion and anxiety among all groups of mice. When mice were subjected to Y maze and NOR tests, the results showed that hM3Dq + Sev + SCH23390 mice exhibited less exploration time in both the novel arm (Figure 5C,D) and novel object (Figure 5E,F) compared with hM3Dq + Sev + propranolol and hM3Dq + Sev + saline mice. And there was no significant difference in the exploration time of the two objects in the training phase of NOR (Figure 5G). These data suggested that the dopamine D1/D5 receptor antagonist but not the norepinephrine antagonist blocked the memory improving effects of LC‐dCA1 activation in CSDS‐induced PND after surgery/sevoflurane.

### 3.5 Dopamine neurotransmitter fluorescence is markedly attenuated in cognitive behavioral tests in CSDS+Sev mice

**FIGURE 6:**
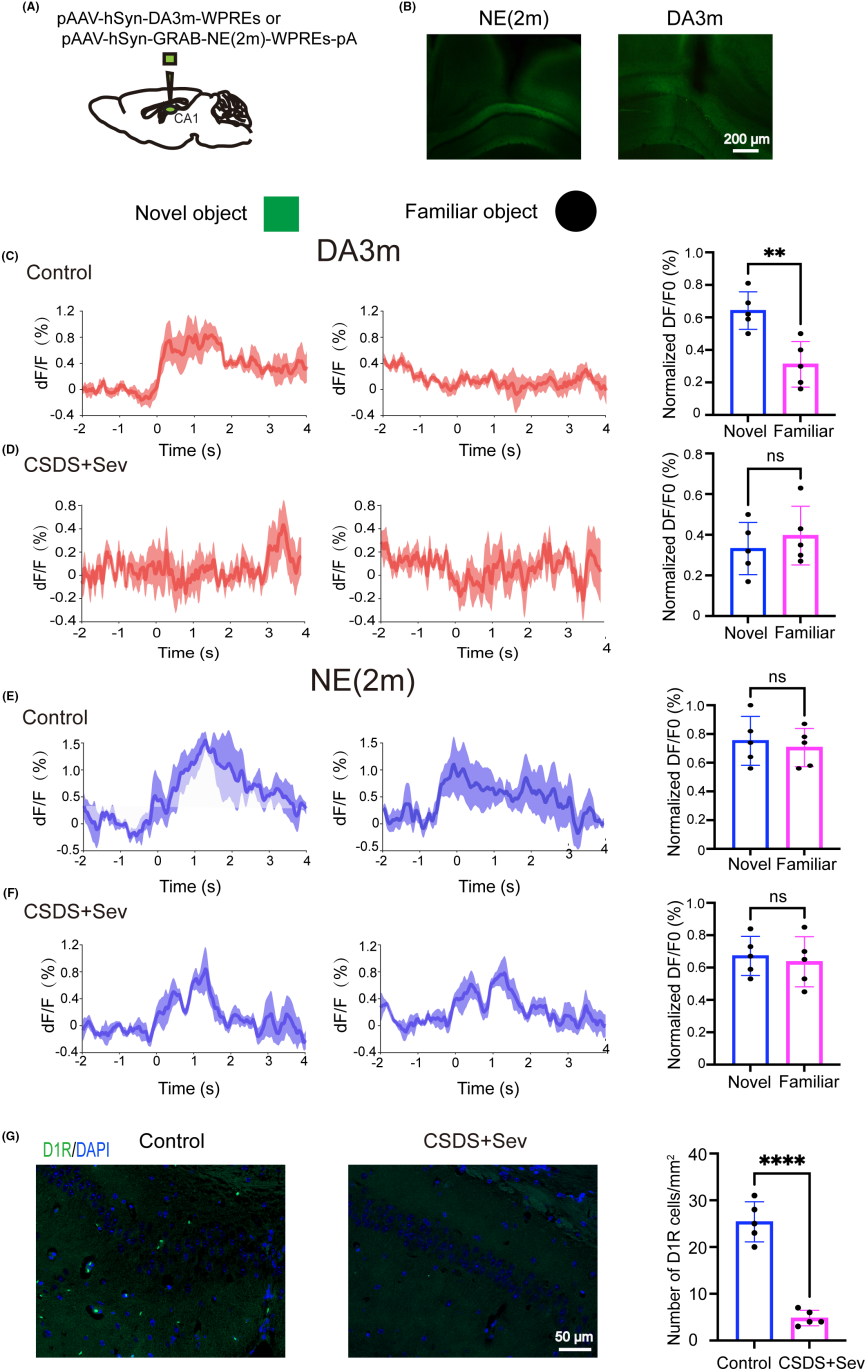
(A) Diagram of viruses injection sites. (B) Representative images of NE (left) and DA (right) probes expression and fiber position in the dCA1. (C) Average DA probe signals when control mice approached the novel (left) and familiar object (middle) and normalized amplitude of the DA probe signals (right, *p* = 0.0055). (D) Same data as (C) in CSDS+Sev mice (*p* = 0.3609). (E) Average of NE probe signals when control mice approached the novel (left) and familiar object (middle) and normalized amplitude of the NE probe signals (right, *p* = 0.7303). (F) Same data as (E) in CSDS+Sev mice (*p* = 0.4559). (G) Representative images of D1R staining in the hippocampus from control (left) and CSDS+Sev (middle) mice, and statistical data of the number of D1R (right, *p* < 0.0001). Unpaired Student's *t*‐test, **p < 0.01, **** < 0.0001, *n* = 5/group. Data are presented as mean ± SD.

As an alternative method to investigate which neurotransmitter is account for depression‐induced PND in adult mice under surgery/sevoflurane, we expressed the fluorescent dopamine or norepinephrine sensors on the dCA1 neurons and monitored fluorescent changes during the NOR test. The genetically encoded dopamine sensor DA3m or norepinephrine sensor NE(2 m) was stereotactically delivered into the dCA1 region (Figure 6A). Virus expression and fiber position are shown in (Figure 6B). Statistical analysis showed that DA3m signals significantly increased once control mice approached the novel object compared to the familiar object (Figure 6C). However, the increase of DA3m signals did not happen when CSDS+Sev mice approached the novel object (Figure 6D). By contrast, the administration of CSDS+Sev (Figure 6F) had no significant effects on NE signals when mice explored the novel object in the NOR test compared with control mice (Figure 6E). Consistent with the results of probe signals, fluorescent staining of dopamine receptors in the hippocampus showed that the densities of hippocampal dopamine D1 receptors in CSDS+Sev mice were significantly reduced compared to the control group (Figure 6G). Overall, these results further demonstrated that reduced dopamine release from the LC may contribute to CSDS‐induced PND after surgery/sevoflurane in adult mice.

## 4 DISCUSSION

This study investigated depression‐induced neurological defects in adult mice after surgery/anesthesia. Our results revealed that CSDS treatment is sufficient to induce PND and hippocampal synaptic defects in adult mice undergoing surgery/sevoflurane. LC‐dCA1pathways played a vital role in this process, specifically, chemogenetic activation of the LC‐dCA1projection displayed protective effects against CSDS‐induced cognitive dysfunction after surgery/sevoflurane. In addition, inhibition of LC‐dCA1 projection partly mimicked CSDS‐induced deficits in learning and memory and hippocampal synaptic impairments. Furthermore, we discovered dopamine release by the LC‐dCA1 pathway plays an important role in depression/CSDS‐induced PND. Overall, these results may provide hints for new intervention targets for alleviating cognitive impairments in patients with depression during the perioperative period.

In the current study, we employed chronic CSDS mouse models but not an acute modeling method (single social defeat stress or lipopolysaccharide administration), because chronic and repeated stress could better simulate human depression factors and characteristics in clinical background.^31^ And it also more precisely reflects mechanisms of affective disorders such as depression. For example, acute social defeat stress has been shown to increase the spontaneous activity of the LC neurons.^32^ However, the activity of the LC system is attenuated after prolonged stress treatment.^22^ Unlike previous studies, our CSDS mice in the first part of experiments did not show a decrease in body weight during depression modeling but remained unchanged. This may be related to the age of the mice used in the first part of our study were 8–10 weeks old and still in the process of gaining weight, as can be seen from the control group mice.

In addition, we chose adult mice as the study subjects, on the one hand, there is little known about the effects of depression on perioperative cognitive performance and the associated mechanisms in the adult stage. On the other hand, aged and young mice undergoing surgical and sevoflurane anesthesia are inherently at higher risk of cognitive impairment,^33^, ^34^ which may confound the role of depression in the development of PND. Several clinical studies in recent years have shown that depression may be an independent risk of postoperative cognitive impairment in old patients.^17^, ^18^ Our findings further demonstrated that adult mice who only received surgery under sevoflurane anesthesia showed no deficits in learning performance and hippocampal damage. However, they were more likely exposed to PND risk when they suffered from chronic depression before.

Furthermore, depression and PND share several common pathogeneses, such as hippocampal dCA1 synaptic alterations dysfunction.^35^, ^36^ Consistent with previous evidence, our results displayed that CSDS led to decreased dendritic complexity and spine density, the sEPSC frequency, and amplitude of dCA1 pyramidal neurons in adult mice with surgery/sevoflurane. It is worth noting that our research did not find any cognitive or hippocampal synaptic alterations impairment in mice receiving depression modeling alone, which is inconsistent with previous reports.^37^, ^38^ This may be due to the fact that we observed cognitive and hippocampal synaptic alterations five days after depression modeling. During this interval period, the pathological damage of depression in adult mice might have been partially restored. Collectively, these data indicated that adult mice with depression prior to surgical/sevoflurane are more likely to cause dorsal hippocampal synaptic alterations impairment, leading to the occurrence of PND.

Previous studies about the LC have focused on its response to stress, however, some recent studies using advanced technologies have highlighted that assessing the whole‐brain projections of LC neurons can lead to a better understanding of their role in regulating different behaviors.^39^ Importantly, the role of spatial learning and memory by regulating neuronal activity in the hippocampus is gradually being valued.^40^ Recent studies have identified that the hippocampus CA1 area in rodents is more preferentially innervated by projections from the LC,^41^ providing a structural basis for these evidence. Furthermore, the deficit of the LC‐dCA1 projections has been validated to participate in the cognitive impairment of depression in humans.^42^ Consistent with these previous findings, our results showed that specifically activating this circuit improved cognitive behavioral performance in surgery/sevoflurane adult mice who suffered from depression before but also had a beneficial effect on dCA1 pyramidal neuronal synaptic alterations. In addition, inhibiting the LC‐dCA1 circuit mimicked the harmful influence of depression on dCA1 pyramidal neuronal synaptic alterations. Interestingly, the silence of the LC‐dCA1 projections only affect the behavioral performance in the NOR test but not in the Y maze test which may be due to the greater role of this pathway in rodent animal for novelty recognition and memory.^43^ Together, our findings suggested that the LC‐dCA1 circuit mediated the vulnerable effects of chronic depression on the PND and hippocampal synaptic alterations of adult mice undergoing surgery/sevoflurane, thus unveiling that complex LC projections are associated with more behavioral function's mechanism.

Despite the significance of the LC‐dCA1 pathway in mediating the vulnerability of cognitive function and dCA1 neurons to surgery/sevoflurane, the precise mechanism by which neurotransmitters released by LC neurons regulate local neural activity within dCA1 has yet to be completely elucidated. According to the synaptic alterations change of dCA1 pyramidal neurons in our study, CSDS appeared to alter the release of neurotransmitters in the LC neurons. In fact, although the LC neurons are the primary source of norepinephrine in the brain, the amount and role of co‐released dopamine cannot be ignored. Especially the role of dopamine in the dorsal hippocampus in mediating different models of learning and memory has been increasingly discovered.^44^ The ventral tegmental area (VTA) is the main source of dopamine in the brain, which has a highly reciprocal connection with the hippocampus.^45^ Therefore, for decades, the VTA has been deemed the main source of hippocampal dopamine that mediates learning and memory.^39^ However, from an anatomical point of view, the ventral hippocampus received more projection from the VTA than the dorsal hippocampus which is required for spatial learning and memory.^46^ By contrast, more and more studies found that the co‐released dopamine from the LC plays a major role in cognitive function.^23^ Similar to previous studies, our current study showed that cognitive‐improving effects were blocked by dopamine D1/D5 receptor antagonist when the LC‐dCA1 pathway was chemogentically activated in CSDS+Sev mice. In contrast, norepinephrine antagonist did not affect behavioral performance. Moreover, we also found that dopaminergic rather than norepinephrinergic signals declined when CSDS+Sev mice explored the novel object in the NOR test. In addition, the densities of D1 receptors were also downregulated by CSDS+Sev modeling. These results uncovered that depression mainly affected the ability of the LC neurons to release dopamine in adult mice undergoing surgery/sevoflurane.

However, there are some limitations in this current study. We exclude female mice in our experiments due to the fact that their cognition and neurotransmitter levels are obviously affected by estrogen fluctuations during the estrous cycle.^47^ But even so, because PND occurs in both sexes, future studies could further investigate the effects of CSDS on PND in adult female mice and the underlying mechanisms. What's more, we only used CSDS‐susceptible mice and did not observe whether CSDS‐resilient mice had cognitive or brain neurotransmitter disturbances during the perioperative period. Finally, the downstream molecular pathways of dopamine‐mediated PND induced by CSDS in adult mice also need to be further explored.

In conclusion, our study has demonstrated that the LC‐dCA1 projection plays a critical role in depression‐induced PND in adult mice with surgery/sevoflurane. Preoperative depression is not only a significant risk factor for the occurrence of PND but also can aggravate its severity and prolong its duration. PND can further induce postoperative depression or aggravate preoperative depression symptoms, significantly increasing patients' postoperative morbidity and mortality and seriously affecting their physical and mental health. Our results may provide hints for a new target for alleviating the adverse effects of PND caused by preoperative depression.

## AUTHOR CONTRIBUTIONS

Kai Zhang and Yonghao Yu contributed to the conception and design of the study. Kai Zhang performed the main experiments and drafted the manuscript. Qianqian Chang performed the histological verification, D1R receptors staining, and data analysis. Feixiang Li was responsible for Gogi staining experiments, and Ran Ding conducted the experiment of the fiber optic part, and helped with manuscript writing. Yun Li helped analyze the results of spines number and dendritic complexity.

## CONFLICT OF INTEREST STATEMENT

The authors declare that there is no conflict of interest.

## Data Availability

The data that support the findings of this study are available from the corresponding author upon reasonable request.
